# Editorial: Versatile Roles for B Cells in Tumor Immunity

**DOI:** 10.3389/fimmu.2022.951933

**Published:** 2022-06-17

**Authors:** Peter Brossart

**Affiliations:** Department of Internal Medicine Oncology, Hematology, Immuno-Oncology and Rheumatology/Clinical Immunology, University of Bonn, Bonn, Germany

**Keywords:** tertiary lymphoid structures, tumor microenvironment, antigen presentation, tumor immunology, cancer, tumor infiltrating B cells, regulatory B lymphocytes, immunotherapy

The present Research Topic titled *”*Versatile Roles for B Cells in Tumor Immunity*”* features 6 articles that highlight the crucial role of B cell elicited effects on the generation of an effective immunity to combat cancer.

The tremendous clinical success of immune checkpoint blockade in the treatment of malignant diseases has confirmed the importance of T cells. However, the role of B cells is increasingly recognized and there is abundant evidence that tumor-infiltrating B lymphocytes (TIL-Bs) have an important and synergistic impact in the control and elimination of malignant cells. They can mediate a powerful anti-tumor immunity that results from antibody production, a unique mode of antigen presentation, release of cytokines and chemokines and their central role in the assembling of the tumor microenvironments (TME). The presence of TIL-Bs has been shown to be a strong prognostic and predictive factor for the efficacy of immune checkpoint blockade or standard therapies (1, 2).

Singh et al investigated in the B16 melanoma mouse model the role of B cells in generating a protective immunity in response to PD-L1 blockade alone or in combination with TLR-7/8 activation. In this model, B cells and the B cell derived chemokine CXCL13 were required to promote the efficacy of anti-PD-L1 alone or in combination with a TLR-7/8 agonist. In contrast, B cells were dispensable for the therapeutic effect of anti-CTLA-4 inhibition. CD40-positive but not CD40-negative B cells contributed to anti-melanoma immunity. Further analyses of melanoma patients’ TCGA data confirmed that the presence of B cells and the CXCL13 chemokine together with CD8+ T cells were strongly associated with improved overall survival.

Conventional B cells arise and mature from the common lymphocyte progenitor lineage in bone marrow, where they undergo V(D)J recombination, resulting in cell surface expression of B cell receptors of the IgM isotype. They migrate from the bone marrow to the spleen and differentiate to marginal B cells that remain in the spleen or follicular B cells that populate the lymph nodes. Upon contact with the cognate antigen, B cells further undergo affinity maturation and class switch recombination that improves antigen specificity. Most of the immature B cells are self-reactive and undergo clonal deletion or receptor editing. However, about 20% of naive mature B lymphocytes exert some degree of self-reactivity and enter the periphery. Peripheral B cell tolerance is mainly maintained by the suppressive effects of CD4^+^ regulatory T cells. Thus, the antigenic repertoires and mechanisms mediating central and peripheral tolerance in B cells differ from T cells and there are several reports demonstrating that TIL-Bs can recognize self-antigens. Human TIL-Bs express HLA class I and II and co-stimulatory molecules and have fundamental implications for processing of tumor derived antigens and the generation of humoral and cellular anti-cancer immunity. In addition, B cells play a central and integral role in epitope spreading (3). Similar to normal B cells TIL-Bs produce various immunostimulatory chemokines and cytokines, including IL-4, IL-6, IFNγ, TNF, C–C motif chemokine ligand 3 (CCL3), IL-2 and GM-CSF. Recent data indicate that tumor tissues contain highly diverse populations of B cells at different developmental states (1, 2).

Yu et al. addressed this topic by extracting the B cell repertoires and populations from 28 different cancer types in the TCGA dataset. They found similar V-gene usage patterns in colorectal and endometrial cancers and significant associations with survival in a subset of tumor types. Although large differences were present in the tumor entities with their B cell repertoire, there were significant associations between the B cell repertoire features and mutation load, tumor stage, and age in a few tumor types.

In the TME TIL-Bs are mostly found in close neighborhood to different T cell populations, NK cells and myeloid cells that can result in the formation of ectopic aggregates of immune cells. These tertiary lymphoid structures (TLSs) display a lymph node-like composition with B- and T cell zones and play a central role in initiating and maintaining of adaptive immune responses (2, 4, 5). Germain et al. analyzed the relationship between TSL-B cells and CD4^+^ T cells in a prospective cohort of 56 NSCLC patients. They observed that tumor-infiltrating T cells showed marked differences according to TLS-B cell presence. A retrospective analysis of 538 untreated NSCLC patients revealed that high TLS-B cell presence counterbalanced the deleterious effect of high Treg density on patient survival, and that TLS-B^high^ Treg^low^ patients had the best clinical outcomes indicating that B cells may play a central role in promoting protective T cell responses. In addition, Wu et al. found that CD20^+^CD22^+^ADAM28^+^ B cells in cancer-associated TLS are involved in the efficacy of check point inhibition and promote the response to therapy. B-cell density was predictive for a response to checkpoint inhibition.

Regulatory B cells (B_reg_) have been described in malignant tumors characterized by the secretion of IL-10. However, according to recent studies regulatory B cells also produce IL-35, granzyme B and TGF-β. B_reg_ can develop from memory B cells, plasmablasts and plasmocytes. In contrast to regulatory T cells B_reg_ cells lack a lineage marker such as forkhead box protein P3 and are defined by their effector molecules. B_reg_ induce T_reg_ polarization, inhibit the effector functions of CD4^+^ and CD8^+^ T cells and antigen-presenting cells. The presence of B_reg_ in tumor tissues has been associated with a poor prognosis (1).

Michaud et al. used a novel IL-35 reporter model to determine the signaling pathways that promote the immunosuppressive effects of regulatory B cells. *In vitro* analysis of IL-35 reporter B cells revealed that the combined BCR and TLR4 signaling is capable of inducing IL-35 expression. However, *in vivo*, B cell receptor activation was central to B cell-mediated suppression and promotion of pancreatic cancer growth. Further analysis revealed protein kinase D2 (PKD2) as a key downstream regulator of IL-35 expression in B cells. Blocking of PKD in B cells inhibited growth of cancer cells and enhanced the effector T cell function upon adoptive transfer into B cell-deficient mice. These data might help to identify novel therapeutic targets to attenuate the function of regulatory B cells.

## Author Contributions

The author confirms being the sole contributor of this work and has approved it for publication.

## Conflict of Interest

The author declares that the research was conducted in the absence of any commercial or financial relationships that could be construed as a potential conflict of interest.

## Publisher’s Note

All claims expressed in this article are solely those of the authors and do not necessarily represent those of their affiliated organizations, or those of the publisher, the editors and the reviewers. Any product that may be evaluated in this article, or claim that may be made by its manufacturer, is not guaranteed or endorsed by the publisher.
